# Predictable Overcompensation in Post-Thinning Stand Dynamics of Canadian Forests: A Synthesis

**DOI:** 10.3390/plants14070982

**Published:** 2025-03-21

**Authors:** Chao Li, Bernard Roitberg, Shongming Huang, Robert Lalonde

**Affiliations:** 1 Canadian Wood Fibre Centre, Canadian Forest Service, Edmonton, AB T6H 3S5, Canada; 2 Department of Biological Sciences, Simon Fraser University, Burnaby, BC V5A 1S6, Canada; 3 Alberta Forestry and Park, Edmonton, AB T5M 3V9, Canada; shongming.huang@gov.ab.ca; 4 Department of Biology, University of British Columbia, Okanagan, BC V1V 1V7, Canada; robert.lalonde@ubc.ca

**Keywords:** compensatory growth, forest productivity, growth and yield, overcompensation, pre-commercial thinning, TAG, TreeCG, thinning theory

## Abstract

Most experiments on thinning effects are based upon short-term outcomes, which has led to a common conclusion that stand gross volume will be reduced after various thinning operations. However, contrary results are emerging from more recent long-term thinning experiments. The well-known biological concept of compensatory growth was introduced to reconcile these opposing results. This synthetic article describes a systematic investigation on overcompensation under the conceptual framework of compensatory growth and consists of the following: (1) empirical evidence of overcompensation in forests; (2) a theoretical proof of the possibility of emerging overcompensation using a life-history-theory based analytical tree adaptive growth (TAG) model; and (3) an empirical data-based tree compensatory growth (TreeCG) model that resembles the growth relationships from natural stands. Our results indicate that (1) overcompensation is an expected common phenomenon across different tree species and geographical regions, and (2) overcompensation can be predicted from at least two different mechanisms: optimal allocation of available energy to growth, reproduction, maintenance and reserves, and redistribution of freed resources from dead trees. Therefore, overcompensation is a predictable phenomenon, and forest managers can make SFM (Sustainable Forest Management) decisions based on their specific management goals. Research recommendations are suggested for next steps.

## 1. Introduction

Enhancing productivity is a primary goal for applied plant and animal scientists in many different research fields. In forestry and silviculture, in particular, it is crucial to elucidate efficient ways of enhancing stand productivity to mitigate disturbance-induced reduction in stand volume (including harvest) to ensure sustainable wood supply and satisfy the increasing demands from human society. Thinning is a common silvicultural tool which has been extensively studied in the past for more-than-two centuries for its impact on post-thinning stand dynamics, and the diverse results has led to some consensus [1,2]; however, some associated phenomena such as increased stand gross volume or overcompensation are still unexplainable by conventional thinning theory. As a result, these phenomena have usually been ignored as “out-of-the-ordinary” or treated as statistical outliers. Nevertheless, such simple conclusions might result in missing opportunities of reaching the goal of productivity enhancement. In this article, we describe a research framework for resolving one such silvicultural puzzle, overcompensation, which provides a pathway for reaching the goal of enhancing stand productivity.

### 1.1. A Silviculture Puzzle

Thinning immediately reduces the stand density and thus its gross volume, and such reduction is unlikely to recover in a short period of time due to the slow growth rate of trees. Therefore, any enhanced stand gross volume in the short-term cannot be expected. These results have been well summarized in the literature (e.g., [1,2]). Several key conclusions reached on thinning include enhanced growth in diameter and height, basal area, and merchantable volume; however, gross volume is usually lower than that from the unthinned sites. These conclusions have been confirmed by numerous short-term thinning experiments worldwide [3] and have driven conventional thinning theory and guided decisions in forestry practices [4]. The drawback of reduced gross volume has been seen as a serious concern by forest managers and practitioners in their implementation of thinning in forestry practice.

Exceptions, however, have also been reported in the literature from time to time. For example, seven decades ago, Steel [5] reported that the gross volume in a pre-commercially thinned Douglas-fir (*Pseudotsuga menziesii* [Mirb.] Franco) stand on the Wind River Experimental Forest in Skamania County, Washington, exceeded that of its unthinned counterparts after 20 years. More than two decades later, Warrack [6] summarized 50-year pre-commercial thinning (PCT) research results from the famous Schenstrom Thinning Plots in BC, Canada [7], showing that after seven major thinnings, there was no difference in growth volumes for whatever treatment was compared with the unthinned control when considering all the site differences. The original Tree and Stand Simulator (TASS) [8] also predicted the stand basal area could be enhanced by PCT and fertilization.

These observations were apparently inconsistent with conventional thinning theory and formed a silviculture ‘puzzle’, i.e., forest productivity cannot be enhanced in the short-term in general, but might be possible from a long-term perspective, despite it being difficult to explain using existing thinning theory. As pointed out by Zeide [2] “in all of the [PCT] trials, the basal area and total volume of the thinned plots is well below that of the unthinned plots, but sufficient time has yet to elapse since treatment to indicate whether the thinned plots will ever catch up to their unthinned counterparts”, and so, revisiting historical PCT sites could provide a fuller picture of the post-thinning stand dynamics. With this approach, Pitt and Lanteigne [9] revisited the historical balsam fir (*Abies balsamea* (L.) Mill.) PCT experimental site in the Green River, New Brunswick, Canada, 42 years after the initial treatments, and found that the stand volumes in treatment sites were about 15% higher than those from untreated sites. Nevertheless, the issue remained uncertain due to insufficient evidence and difficulties in explanations and predictions in practical applications.

### 1.2. A Research Framework

In analyzing a 40-year PCT and fertilization experimental dataset from Shawnigan Lake, BC, Canada, Li et al. [10] introduced the concept of compensatory growth (CG) from general biology to explain the diverse patterns in post-thinning stand dynamics. CG is a common phenomenon widely observed in animals and plants [11] but not previously reported in forests. Li et al. [12] defined CG in forestry as “a change in growth rate, usually positive, in a forest stand following a disturbance that reduces biomass and/or individuals from the population” and provided a number of examples and research progress in different fields. CG is a process that consists of a spectrum of states ranging from under-compensation (stand gross volume from a thinned site is lower than that of an unthinned counterpart site), exact compensation (stand gross volume from a thinned site is equal to that of an unthinned counterpart site, and can also be called compensatory-induced-equality, CIE), and overcompensation (stand gross volume from a thinned site is higher than that of an unthinned counterpart site) over time. Conventional thinning theory has described under-compensation short-term post-thinning stand dynamics across species and geographical regions well, in addition to how increased stand productivity in long-term post-thinning stand dynamics falls in terms of overcompensation, this being the other end of CG spectrum. The work described below focuses on overcompensation that represents enhanced stand gross volume. CG is a special case of growth response that indicates how trees respond to a disturbance in their environment, i.e., CG needs to be triggered by a disturbance of reduced biomass. A possible way of inducing expected post-thinning stand dynamics, such as overcompensation, could be manipulated by altering the disturbance in terms of its timing and intensity, as well as the rules of thinning [11].

To examine whether overcompensation is possible in forest stand productivity enhancement in post-thinning stand dynamics, a combined empirical data analysis and modeling approach is employed. The empirical data analysis is employed to compile historical long-term silvicultural datasets and identify the stand growth patterns after thinning treatments. This work determines whether the reported overcompensation is singular/unique or a common case. Thus, we compiled five published long-term thinning datasets for different major commercial tree species across Canada and concluded that overcompensation is a common phenomenon that appeared in all five datasets. If overcompensation can be seen as a common phenomenon in Canadian forests, there is a need to identify the possible underlying mechanisms for predicting its occurrence to support decisions in forestry practices.

Since any field experiments in forests will take a long period to complete, it makes sense to apply a modeling approach to enhance the exploration of logical consequences of possible mechanisms of overcompensation. In this regard, we aimed to determine if any existing theory in biology/ecology could be applied to our conceptual framework. Any positive answer would confirm that overcompensation is an expected outcome under specific conditions. We also address the question as to whether existing stand growth relationships obtained from natural stands can be employed to predict the occurrence of overcompensation and thus support the decisions in sustainable forest management (SFM).

### 1.3. Objectives and Presentation Arrangement

This article presents a systematic investigation on whether overcompensation is a predictable phenomenon in post-thinning stand dynamics, with the specific objectives of (1) summarizing the empirical evidence of overcompensation in Canadian forests; (2) explaining a theoretical proof that overcompensation can be expected in long-term post-thinning stand dynamics; (3) providing an example of published stand growth relationships that can be used to predict the occurrence of overcompensation; and (4) discussing the pros and cons of overcompensation and recommending the next steps in research. We describe the results associated with these objectives next.

## 2. Empirical Evidence

### 2.1. Long-Term PCT Data

**Figure 1:**
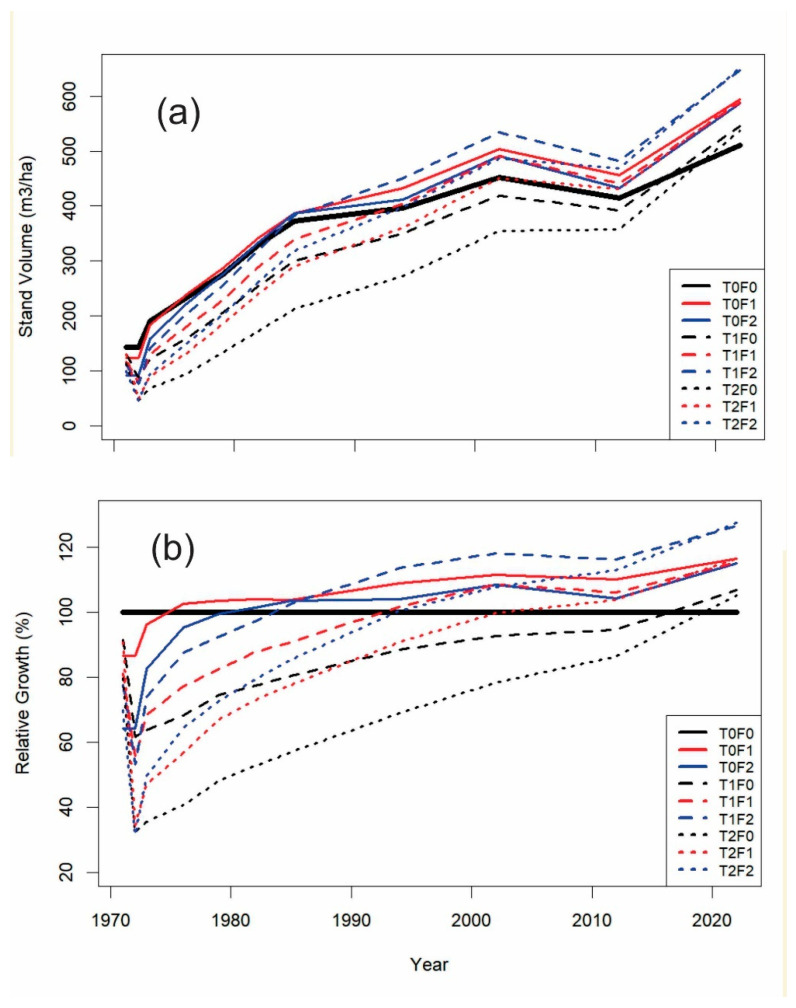
Observed 50-year post-thinning stand dynamics from Shawnigan Lake trial represented in (**a**) stand gross volume over time, and (**b**) growth of stand gross volume, relative to unthinned control, over time under different treatments compared to no thinning, no fertilizer treatments (T0F0).

The research took place under the premise that should overcompensation be a truly common phenomenon in forests, there should be considerable empirical evidence demonstrating its occurrence. In searching the empirical evidence, we started by analyzing a Douglas-fir PCT and fertilization experimental dataset from the west coast of Canada that spans 40 years, near Shawnigan Lake, British Columbia (lat. 48°38′13″ N; long. 123°43′16″ W). Douglas-fir is a major commercial tree species with a long lifespan of at least 500 years, sometimes exceeding 1000 years in BC and the Pacific Northwest Region. It has also been a major plantation species for more than half a century with an extensive history of research activities. The Shawnigan Lake trial was established with a 24-year-old stand, with a unique factorial experimental design that enables researchers to understand the effect of various combinations of PCT and fertilization treatments on stand dynamics [13]. The treatments included three levels of PCT (0, 1/3, and 2/3 removals named as T0, T1, and T2) and three levels of fertilization (0, 224 kg/ha, and 448 kg/ha fertilizers named as F0, F1, and F2), with all stands equally managed otherwise. Four plots were run for each of the nine treatments of combined PCT and fertilization. The other feature of this experiment is the multiple remeasurements over time, which allow for the display of a full picture of the stand dynamics, before and right after initial treatments, 3, 6, 9, 12, 15, 18, 24, 32, 40, and 50 years after initial treatments. The most recent remeasurement was conducted in 2022. Figure 1a shows the estimated stand volumes from different treatments over time. As estimated, absolute stand volumes may vary when using different volume equations, even with same measured DBH and H [14]; these volumes are transformed into relative values against the volumes of control plots using the following equation to reduce/eliminate such differences, and are presented in Figure 1b:

(1) $$RG(\%)=Vol_{Treat}/Vol_{Control}\times 100\%$$

Figure 1a shows that stand volumes are significantly reduced as a result of thinning operations, but they gradually catch-up with unthinned (T0F0) sites over time and eventually exceeded that of the unthinned, unfertilized controls (T0F0) in the long run. This clearly demonstrated how the concept of compensatory growth can characterize diverse growth pattens, i.e., compensatory growth is a gradual process, from under compensation right after thinning (stand volume of thinned stands is lower than that of control) to gradual catch-up growth to exact compensation (stand volume of thinned stands becomes equal to that of control), and eventually to overcompensation after that (stand volume of thinned stands becomes higher than that of control).

As stand volume is represented by relative growth in Figure 1b, different states of compensatory growth can be seen more clearly: a stand volume of 100% for T0F0 is represented as a horizontal line, and any stand volume under the 100% line indicates under compensation; any stand volume that intercepts with 100% represents exact compensation, and any stand volume above 100% line denotes overcompensation. In T0F1 (light fertilization without thinning), for example, a greater performance than the control can be seen starting at 5 years after the treatment. In T2F0 (heavy thinning without fertilization), as another example, overcompensation can only be seen at nearly 50 years after the treatment.

**Figure 2:**
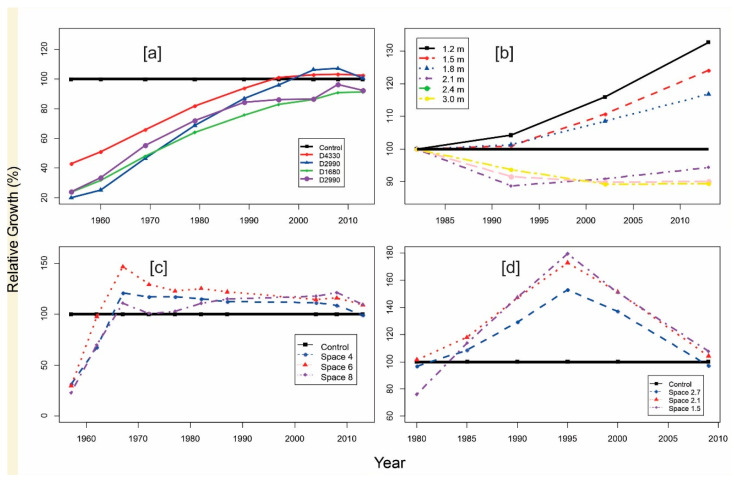
Diverse overcompensation patterns from four long-term thinning datasets across Canada. Values exceeding 100 (the control line) are overcompensation; (**a**) Lodgepole pine in Rocky Mountain foothills, Alberta; (**b**) red pine in Petawawa, Ontario; (**c**) balsam fir in Green River, New Brunswick; and (**d**) hardwood species in Flat Top, New Brunswick.

Figure 2a shows the post-thinning stand dynamics of lodgepole pine (Pinus contorta Douglas) from a long-term experiment of stand density management through PCT, expressed as relative growth. Lodgepole pine is a common tree in western North America, and the provincial tree for AB. The experiment was established in 1954 and located at the MacKey, on the eastern slopes of the Canadian Rocky Mountains, Alberta (lat. 53°32.7″ N; long. 115°32.3″ W). The original purpose of the experiment was to determine whether the PCT of lodgepole pine could improve merchantable volume and quality at a young age and, in turn, shorten rotation and increase annual allowable cuts [15,16]. The experiment was implemented on plot sizes of 0.08 ha each in block A, B, and C, and 0.30 ha in block D, measured for DBH at 1.3 m for all living trees, with PCT treatments that reduced initial stand densities ranging from 9762 to 11,887 stems per ha, to 4330, 2990, 1680, and 750 stems per ha, on 22-year-old stands. The stand conditions were measured in 1954, 1960, 1969, 1979, 1989, 1996, 2003, 2008, 2013, 2018, and 2023. However, only the data collected before 2018 were used in the current analysis. The relationship between thinning intensity and compensatory response is inconsistent; however, it appears that too severe thinning may prevent stands from ever catching up or surpassing control plots. We will return to this point in the modeling section below (Section 3).

Figure 2b shows the cumulated stand gross volume of red pine (Pinus resinosa Aiton) from a commercial thinning experiment, established in 1953 and located in the Petawawa Research Forest in central Canada, Chalk River, Ontario (lat. 46°00′0″ N; long. 77°26′0″ W) [16,17], expressed in relative growth. The spacing experiment was established on abandoned farmland, with a factorial design of six initial spacings of 1.2, 1.5, 1.8, 2.1, 2.4, and 3.0 m randomly allocated to 10 stands. In 1962, two 0.08–0.101 ha permanent sampling plots were established for each spacing and measured periodically, and the DBH and H before each of the thinning entries were measured for these permanent sampling plots. DBH was measured for every tree in each sample plot, but H was only measured for a subsample of trees (19–76% of live stems). The commercial thinning was performed repeatedly in 1992, 2002, and 2013 to bring BA down to the same original residual BA target value of 37.9 m^2^/ha.

Figure 2c shows the post-thinning stand dynamics of balsam fir from the Green River watershed, located in eastern Canada, in the extreme northwestern corner of New Brunswick (lat. 47°46′ N; long. 68°17′ W), expressed as relative growth. The area contains approximately 100,000 ha of balsam fir-dominated forests, and the experiment was aimed at studying the effects of PCT and vegetation management on the growth, yield, and value [18,19,20,21]. The experiment included three nominal spacings of 4 ft (1.2 m), 6 ft (1.8 m), and 8 ft (2.4 m) compared to an unthinned control in six replicate blocks. Forty-eight permanent sample plots were measured every five years between thinning and 30 years post thinning. The study site was revisited in 2004 and measured at 43 years post thinning and with PSPs reevaluated in 2008 and 2013.

Figure 2d shows the results from a spacing experiment at Flat Top Mountain near Pokiok, New Brunswick (lat. 45°54′04.7″ N; long. 67°16′39.6″ W), established in 1979 for determining the proper spacing to shorten the pulpwood rotation that meets hardwood kraft mill requirements, by the Valley Forest Products Ltd. (Langley, BC, Canada), Woodlands Division of the Ste. Anne Nackawic Pulp Co. Ltd. (Nackawic, NB, Canada) [22]. The main hardwood species include beech (*Fagus grandifolia* Ehrh.), white birch (*Betula papyrifera* March.), yellow birch (*Betula alleghaniensis* Britt.), red maple (*Acer rubrum* L.), sugar maple (*Acer saccharum* L.), and ironwood (*Ostrya virginiana* (Mill.) K. Koch). Three spacings, 5 × 5 ft (1.5 m), 7 × 7 ft (2.1 m), and 9 × 9 ft (2.7 m), were compared with an untreated control in 0.2-acre (0.08-ha) plots in a 4 × 4 Latin square design. Within each plot, a 1/10-acre (0.04-ha) measurement plot was established, and all trees were tagged for remeasurement in 1980, 1985, 1990, 1995, 2000, and 2009.

### 2.2. Diverse Patterns

A common feature of the above five empirical datasets is that overcompensation occurred at certain periods after the thinning treatments. However, the dynamics of overcompensation are diverse in terms of timing and amplitude.

In the Douglas-fir case (Figure 1), the slow speed of CG resulted in a gradual transition in status from under compensation right after the thinning operations, to exact compensation, and eventually to overcompensation (up to ~130%). With increasing thinning intensity, the timing to reaching exact compensation can be significantly delayed (ranging from 15 to over 45 years). On the other hand, increased fertilization level can reduce the period required to reach exact compensation.

In the case of lodgepole pine (Figure 2a), it took more than 40 years to reach exact compensation, and some plots remained in an under-compensation state without reaching transition status. An interesting point is that two plots with same target stand density of 2990 stems per ha displayed different patterns: one reached overcompensation and the other did not. This suggests that individual difference in response to thinning might be highly variable.

For balsam fir (Figure 2c), a faster transition from under compensation to exact and overcompensation was observed, but then compensation declined to a lower level (still higher than that of unthinned sites), followed by maintenance at that level over time. The 6 ft spacing treatment generated the highest overcompensation of ~150% at 10 years after thinning treatments.

For the hardwood species (Figure 2d), a pattern similar to balsam fir appeared; however, it is still uncertain as to whether this lower level of overcompensation will be maintained, which will be confirmed when the most recent remeasurment data become available. The 1.5 m spacing treatment generated the highest overcompensation at 15 years after initial treatments.

For the case of red pine (Figure 2b), it appears that overcompensation can be observed at the spacings of 1.2, 1.8, and 2.1 m, but not at the larger spacings of 2.1, 2.4, and 3.0 m.

### 2.3. Possible Influencing Factors

From a perspective of forestry practices, identification of possible influencing factors for diverse overcompensation patterns is important for assisting managerial decision making. The following is a preliminary summary from the above datasets (see Figure 1 and Figure 2):

- Tree species: The lifespans for different species might be the first notable factor. Species with a long lifespan tend to require longer periods to reach the CG transition status, such as with Douglas-fir, which has a longevity of over 800 years. Species with a short longevity (e.g., 150-year lifespan for balsam fir and hardwoods) might be expected to reach overcompensation in a shorter period.
- Site quality: Plots with a poor site quality (e.g., Douglas-fir experimental site with a poor site index) tend to require long periods to reach overcompensation. On the other hand, plots with a high site quality tend to have a fast CG status transition, like with the balsam fir experimental site, which has a very high site index. Compared with the same species and Acadian Forest region in Maine, US, but which has a low site quality, complete overcompensation was not observed 32 years after thinning operations [23].
- Physical environmental conditions: Sites with poor climatic conditions could delay tree growth and thus require long periods to reach overcompensation, such as with lodgepole pine growing at Rocky foothills, where the winter tends to be longer than that for Douglas-fir growing near the Pacific coast, with warm winters.

The stand structure, the mixing regime, and the species’ light demands are potential influencing factors for stand dynamics. Therefore, they could also be considered potential influencing factors for diverse overcompensation patterns. But since they are primarily driven by species composition, site quality, and physical environmental conditions, they are embedded in the aforementioned factors and not separately addressed further.

Given the clear yet highly variable evidence for stand response to thinning, there is a clear need for a general theory that can account for the aforementioned phenomenon of compensatory dynamics, i.e., simply reducing stand density is not sufficient to generate overcompensation. We expect that the inherent differences in life history [24] and resource acquisition are required for such a unified theory. In the next section, we present such a theory based on life-history.

## 3. A Theoretical Proof of Concept

Despite the fact that the concept of compensatory growth can explain overcompensation in the long-term and post-thinning stand dynamics well, and thus reconcile the controversial observations from both short- and long-term silviculture datasets, it is critical to identify the underlying mechanisms that lead to overcompensation. We explored a possible mechanism from the life-history theory (LHT), the most cited ecological theory in the literature [25], which is a framework in evolutionary biology that elucidates how natural selection influences patterns of growth, reproduction, and survival across an organism’s lifespan. The LHT is based on the premise that organisms generally face trade-offs during their life, arising from various causes, including energetic, physiological, developmental, environmental, and genetic constraints, which determine the patterns of survivorship and reproduction we observe in nature [26]. When applied to trees, LHT can help explain how different species adapt their growth and reproductive strategies to their environments.

LHT is applicable to organisms with either short or long lifespans. Compensatory growth (CG) is a special case of growth response, triggered by some sudden and catastrophic events such as thinning. The CG process in short-lifespan species can happen fast or very fast, and experiments that generate CG are usually easy to implement, with outcomes which are consistent for a single status (either under, exact, or overcompensation) at the time of measurement (see [12] for a summary of examples). However, it is generally difficult to elucidate how the process plays out over time because the short time scale can obfuscate growth dynamics. For long-living species such as trees, despite the technical difficulties of working with such slow-growing organisms, these species are excellent model species for documenting the CG process over time, providing a fuller picture for investigating the CG process because their ontogeny unfolds slowly, and so it is easy to characterize the process for these organisms.

### 3.1. The TAG Model

Roitberg et al. [27] described a simple, theory-driven simulation model called Tree Adaptive Growth (TAG), which explores the thinning-driven stand dynamics based on the principle of LHT for individual trees, in the sense that individual trees allocate mobilized energy to (1) vegetative growth, leading to morphological structure build-up, (2) seed production for reproduction, reflecting the evolutionary fitness to be maximized, (3) maintenance of the morphological structure that is often assumed as default, and (4) storage in reserve for meeting future allocations that is important for multi-growing-season species. The trade-off facing an individual tree is how to optimize the allocation to these processes to achieve maximized lifetime fitness under various environmental conditions.

The key to implementing this LHT-based approach is state-dependence, which is differentiated from the conventional age-dependent growth prediction [11] in the sense that tree growth is determined by yearly changes in internal and external/environmental conditions/states, such as the internal states of tree size (S), age (A), and the energy reserve (R), and the external states of the available level of nutrients or competitive ability (ψ), and fluctuations in weather variables (W). This approach assumes that changing annual growth is an adaptive response of trees to their environmental conditions and also implies that simulated tree growth curves may not be as smooth as predicted by age-dependent relationships.

The TAG model employs the following four basic assumptions:

1. Innate tree growth follows a sigmoidal pattern [28];
2. Tree growth strategies have evolved to maximize lifetime reproductive success according to the principles of evolutionary ecology [29];
3. Individual trees will acquire resources and modify their investment in growth in a state-dependent manner, according to (S), (R), and (A);
4. There are no genetic constraints on the above, i.e., it is assumed that selection onphenotypic variation translates directly into selection on heritable variation in the population [30].

The model uses a modified logistic submodel to characterize the potential growth increment for a tree of size (s) as follows:

(2) $$\gamma =(\rho -1)(1-{\left(s/s_{\max}\right)}^{\theta }(e^{-\zeta y})$$

where γ is energy harvesting, ρ is the intrinsic growth rate, Smax is the maximum mass that an individual tree can realize, θ is a shape parameter, and ζ is a senescence term that describes decreasing energy harvesting with age. For any given year, a resource allocation strategy is determined according to the combination of the current three state values (s, r, a) of individual trees. In each year, a focal tree will mobilize reserves for growth and reproduction, at a rate of alpha (α).

(3) $$\alpha =(r-R_{\min})/(1+e^{-(w_{1}s+w_{2}a)})$$

where R_min_ is the minimum reserve value for a tree of size (s), and w values are weight constants for the state variables. The α is expected to increase with size, reserves, and age up to some maximum that is not to exceed its critical reserve state value (R_crit_), which is the minimal level required to maintain metabolic function. Then, the proportion of mobilized reserves that go to reproduction versus growth is calculated as (β):

(4) $$\beta (s,r,a)=\left\{\begin{array}{c} 1-e^{-(w_{3}s+w_{4}a)}\;\mathrm{if}\;r>\pi \\ 0\;\;\;\;\;\;otherwise \end{array}\right.$$

where π is the start-up cost for initiating reproduction and the w values are weight constants for size and age states. Two effects of size are assumed: (i) small trees have small reserves such that r will rarely exceed π, meeting the β = 0 condition, and (ii) small trees are inefficient at producing seeds; thus, the size weight (w_3_) is set low. Furthermore, older trees should strongly bias their mobilized reserves toward reproduction because future discounting will offset small gains from growth, particularly if older trees are large; therefore, a small value for (w_4_) is employed.

Finally, the allocation of harvested energy to growth versus reserves is defined as (φ):

(5) $$\phi =\left\{\begin{array}{c} {}[1/(\phi_{s}+\phi_{r})]\phi_{s}\;\mathrm{if}\;r^{\prime }<s^{\prime }R_{\max}\;\mathrm{and}\;r^{\prime }<s^{\prime }R_{\min}\;\\ 1-[(r-\alpha +\gamma -sR_{\max})/\gamma ]\;\;\mathrm{if}\;r^{\prime }<s^{\prime }R_{\max}\\ 1-\{[(s+\gamma )R_{\min}-r^{\prime }]/\gamma \}\;\;\mathrm{if}\;r^{\prime }<s^{\prime }R_{\min} \end{array}\right.$$

where $\phi_{s}=1-(s-s_{\min})/s$, $\phi_{r}=1-[r-(R_{\min})]r$, $s^{\prime }=s+\gamma [1/(\phi_{s}+\phi_{r})]\phi_{s}+\alpha (1-\beta )\xi$, and $r^{\prime }=r+\gamma [1/(\phi_{s}+\phi_{r})]\phi_{s}-\alpha$ are potential updated values for size and reserves, respectively, and ξ is the efficiency in mobilizing reserves to the structure.

The trade-off between growth and reproduction is based on the satisfaction of three conditions: (i) sufficient reserves remain after metabolic costs; (ii) reproduction start-up cost is paid; and (iii) allocation to reserves does not exceed the maximum reserve level after adjusting for γ. Otherwise, φ is adjusted to meet those minimum and maximum reserve levels, respectively. The rationale for the φ decision is that as trees increase in size, their growth rate decelerates; thus, there is little gain from further investment in growth vs. the buildup of reserves for reproduction [31]. The above three state-dependent strategies can then be summarized as follows: (i) investment in growth when that leads to accelerated returns, but only when reserves are sufficiently high to avoid starvation (i.e., metabolic reserves fall below critical level), and (ii) investment in reproduction when trees are large and growth is constrained, when it is safe to do so because of the greater return on investment for the latter. Also, note that future discounting means that future reproductive returns from current investment in growth will be constrained for older and larger trees.

The LHT logic that provides a mechanism of explaining overcompensation is that trees in thinned stands acquire more energy due to reduced competition and that they invest a greater proportion of this greater acquisition than control trees because they will not risk death through starvation during bad years. Control trees are forced to invest conservatively in growth, whereas thinned trees are no longer constrained by LH parameters.

Detailed implementation methods are documented in [27]; below, we summarize the main results from the model simulations in the next section.

### 3.2. Overcompensation Prediction

**Figure 3:**
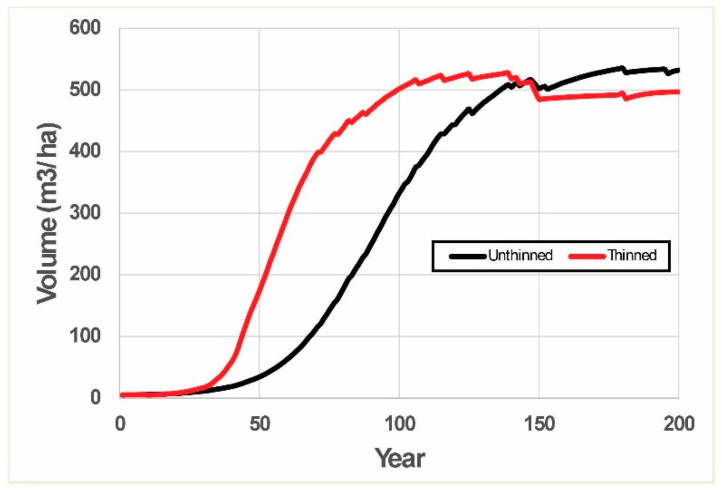
Mean stand volume over time from 10 stochastically simulated TAG stands that initially comprised 100 trees each that were either subjected to pre-commercial thinning of 30% at year 10 (red line) or left unthinned (black line). Simulations were run using C++. Growth of individual trees was based upon stochastically determined weather conditions, relative competitive value of individual trees, and investment by those trees in growth. See [27] for details.

Predictable overcompensation is represented by the two series of simulations in thinned versus unthinned stands. In each series, 10 replications (plots), with 100 trees in each plot, are performed for a hypothetical plantation stand, and the mean stand gross volume is plotted as a representative. In the unthinned stand, trees are allowed to freely grow until the end of simulation (200 years in this case). For the thinned stand, a pre-commercial thinning (PCT) is conducted at year 10 with 30% of the smallest trees removed. The basic pattern of stand dynamics is shown in Figure 3.

This result supports the notion that overcompensation should be a common outcome in post-thinning forest stands when the stand growth over time is sigmoid shaped. The exact growth curve could vary under different environmental conditions such as competition pressures [27]. This theoretical proof should also be generally applicable to other plant and animal species.

The TAG model allowed theoretical exploration of how different thinning operations (a combination of timing, intensity, and rules of thinning) could affect post-thinning stand dynamics. These theoretical explorative investigations indicated that (1) overcompensation in post-thinning stand dynamics is an expected outcome according to LHT, rather than being a statistical outlier, and (2) diverse patterns of overcompensation could be expected, depending on how the thinning operations are implemented. Therefore, to take advantage of overcompensation, forest managers and practitioners need to calibrate the model to their own area under management, and the optimal thinning strategy needs to be determined based on tree species, geographical regions, and physical site conditions.

With the above theoretical demonstration of expected overcompensation in post-thinning stand dynamics, overcompensation should be predictable through assembling available growth relationships. In the next section, we describe a model as an example that employed published growth relationships from natural stands.

## 4. Growth Relationship-Based Overcompensation Prediction

As mentioned previously, CG is a special case of growth response, triggered by a disturbance that causes biomass reduction. Therefore, we examined the premise that if overcompensation is an expected outcome in post-thinning stand dynamics, it should be predictable from the quantitative growth relationships obtained from natural stands. Li et al. [32] described one such model called Tree Compensatory Growth (TreeCG), and the underlying mechanism is that a forest will redistribute the freed resources such as space and nutrients from dead trees to the remaining survived trees to reach a full utilization of the available resources.

### 4.1. The TreeCG Model

Different from the conventional increment of stand volume over time directly, the focus of TreeCG is to take into account the state of the mean resources available for individual trees, which changes with the changing stand density, and simulates the annual increments of DBH and H that facilitate the calculation of gross and merchantable volumes, as well as the estimation of various forest products and values [14]. In other words, TreeCG goes a step deeper, looking into the published growth relationships and assembling them in a non-traditional way.

TreeCG simulates how major mortalities affect stand density for yearly time steps. In addition to the natural mortality of trees each year, stand density is also influenced by thinning operations. Correspondingly, increased annual growth increments of DBH and H can usually be displayed following the thinning year, as released growth that will be damped over time. Therefore, the total annual growth increments can be split into the sum of normal growth (without thinning) and released growth (after thinning). As a result, the key relationships required for simulating the dynamic annual increments are the normal growth increments and the released growth increments. If overcompensation took place in nature, one should be able to predict its occurrence through the growth relationships from natural stands.

As common sense tells us that trees generally grow taller in high-density stands than in low-density stands, and thinning is a common forestry operational tool for reducing stand density to achieve the goals of stand density management, the relationship between stand density and H is essential for investigating the thinning effect on stand dynamics. Such information could generally be derived from the variable-density yield tables, and the one published [33] for natural stands of lodgepole pine in Alberta, Canada, was used in developing the TreeCG model:

(6) $$\begin{array}{l} \log_{10}H=1.0688-0.00276672\times Age+0.717927\log_{10}Age\\ -0.0000371084\times Stems-0.132622\log_{10}Stems \end{array}$$

where Stems is the number of stems with a DBH larger than 0.6 inches of outside bark per acre. By converting the units in the formula from the British system into metric system, we were able to calculate the H of each tree at a given stand density and hence the corresponding DBH through the softwood equation [34] with species-specific parameters for lodgepole pine in Alberta:

(7) $$DBH=\{b_{1}/b_{2}\times {\left[1-\exp(-b_{1}\times b_{2}\times (H-1.3)]\right\}}^{1/b_{3}}$$

**Figure 4:**
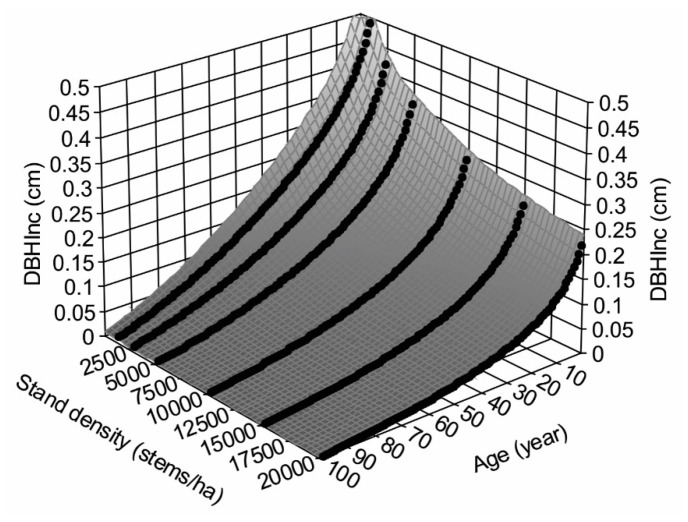
Response surface of annual increments of DBH, DBHInc, at different stand ages and stand densities based on variable density yield table [33] (redraw from [32]).

As such, the annual increment of DBH, DBHInc, can be calculated as below:

(8) $$DBHInc_{t}=DBH_{t+1}-DBH_{t}$$

where the subscript t and t + 1 indicate the measured variables at the year t and t + 1, respectively; a response surface of how the DBHInc decreases with increasing tree age and stand density was constructed (Figure 4), which is consistent with the common observations of faster growth of trees in young stands.

Applying the surface smoothing techniques, the DBHInc at any given age and density without thinning can be estimated as below:

(9) $$\begin{array}{l} z=a+b\ln(x)+c\ln(y)+d{\left(\ln(x)\right)}^{2}+e{\left(\ln(y)\right)}^{2}+f\ln(x)\ln(y)+g{\left(\ln(x)\right)}^{3}\\ +h{\left(\ln(y)\right)}^{3}+i\ln(x){\left(\ln(y)\right)}^{2}+j{\left(\ln(x)\right)}^{2}\ln(y) \end{array}$$

where x is tree age (year), y is stand density (stems/ha), z is DBHInc (cm), and a, b, c, d, e, f, g, h, i, and j are parameters.

For tree growth with thinning at the year of thinning, the altered tree DBHInc_actual_ can be decomposed as the sum of DBHInc of stand density before thinning, DBHInc_natural_, and the released growth, DBHInc_natural-new_, of stand density after thinning:

(10) $$DBHInc_{acturl}(cm)=DBHInc_{natural}(cm)+DBHInc_{natural-new}(cm)$$

where the subscripts natural and new indicate the stand density before and after the thinning operation. To calculate DBHInc_actual_ (cm), we need to have both DBHInc_natural_ (cm) and DBHInc_natural-new_ (cm). DBHInc_natural-new_ (cm) is the released growth of DBH induced by the thinning operation. To simplify the DBHInc_natural-new_ (cm) calculation, DBHInc_natural_ (cm) and DBHInc_new_ (cm) are presented in a relative sense (with regard to an assumed maximal stand density of 20,000 stems/ha for lodgepole pine), and in this case, as RDBHInc_natural_ (%) and RDBHInc_new_ (%), respectively:

(11) $$DBHInc_{natural}(\%)=DBHInc_{natural}(cm)/DBHInc_{20,000}(cm)\times 100$$

(12) $$DBHInc_{new}(\%)=DBHInc_{new}(cm)/DBHInc_{20,000}(cm)\times 100$$

where $DBHInc_{actual}(cm)=DBHInc_{natural}(cm)\times [1+RDBHInc_{natural-new}(\%)]$, and $RDBHInc_{natural-new}(\%)=\frac{RDBHInc_{new}(\%)-RDBHInc_{natural}(\%)}{RDBHInc_{natural}(\%)}\times 100$.

These equations allowed the estimation of DBHInc_actual_ (cm) directly from the stand densities before and after thinning. The same surface smoothing Equation (9) is applied, where x is tree age, y is stand density, and z is DBHInc_actual_ (cm). In other words, RDBHInc_natural-new_ (%) can be obtained by calculating two stand densities, before and after thinning.

The response surface of H after the released growth, corresponding to DBHInc_actual_ (cm), can then be obtained using the provincial equation for lodgepole pine [35]:

(13) H=1.3+35.7547/{1+exp[3.8234−1.2824ln(DBH)]}

The above adjustments allow for a much simplified calculation of the annual increments of DBH and H, and easy implementation in the TreeCG model. See [32] for parameters and other details.

### 4.2. Model Validation

If the way of assembling growth relationships is reasonable, the logical consequences of the model simulations should reflect the observations from the field experiments, i.e., the model will be able to be validated against empirical observations. A qualitative model validation was conducted accordingly through comparing the simulated forest growth patterns to a long-term silvicultural experiment, the Shawnigan Lake PCT and fertilization dataset described in the above empirical evidence. The focus of the comparison is not the matching of absolute values, because the relationships employed in the TreeCG simulation were not specifically from the Douglas-fir forests.

The initial stand density was set as 7500 stems/ha, and the simulation results were evaluated for the total gain over a planning horizon of 100 years, which accounted for the sum of the immediate gain from the thinning operation and the gain from the remaining trees at the year 100. Thinning operations were implemented at two different stand ages, of 30 (representing an early partial mortality) and 60 (representing a late partial mortality) years old. A control without thinning was also implemented. The partial mortality was represented by the intensity of thinning, which was set as a reduction of 33% (as a light mortality) and 66% (as a heavy mortality) of the total number of trees. If this premise were true, the simulated CG capacity could differ under early and late partial mortality, as well as light and heavy mortality.

**Figure 5:**
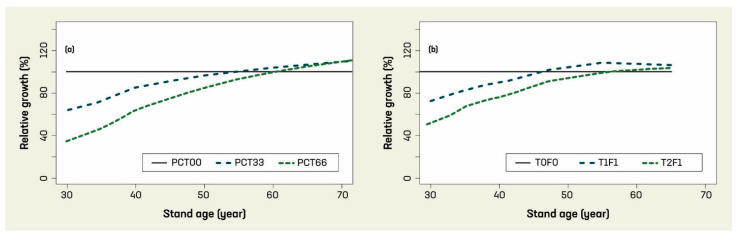
TreeCG model validation: (**a**) simulated and (**b**) observed relative growth in stand gross volume under different pre-commercial thinning (PCT) treatments.

The model output includes the stand conditions (DBH and H of each living tree) at initialization and every 5-year interval until 100 years, and harvested wood at the year of thinning. The simulation results are presented in relative growth terms to reflect the patterns in post-thinning stand dynamics, as in Figure 5.

Figure 5a shows the gross stand volume after experiencing an early thinning at year 30 under three levels of intensity (i.e., 0, 33%, and 66% removal for the initial stand density of 3950 stems/ha, indicated by PCT00, PCT33, and PCT66, respectively), in which partial mortality first reduced stand gross volume and then the stand gross volume gradually catches up with the gross volume of the control stand, and eventually exceeds the control and results in overcompensation. The heavier thinning (PCT66) resulted in higher overcompensation than that of the lighter thinning (PCT33). The trends displayed in this series of simulations appeared consistent with those observed in the measurements from the Shawnigan Lake trial (Figure 5b). This indicates that our simulation results are validated qualitatively by the observations from this long-term PCT and fertilization experiment, and the overcompensation is a predictable phenomenon.

## 5. Discussion

Forests are invaluable resources for human society in the commercial (productivity such as biomass and wood products) and associated non-commercial (ecosystem services such as for carbon, biodiversity, habitat, hydrology, and air quality) senses. As such, a key focus of forest management is ensuring the sustainable use and conservation of forest resources [36]. Here, we evaluated the potential for enhancing overcompensation via pre-commercial thinning, an approach that is supported by empirical studies and modeling exercises. Further, we showed that such overcompensation can vary dramatically among tree species based upon inherent growth rates and site characteristics. Regardless, overcompensation can have a positive impact in reaching the goals of SFM, through providing a new way of enhancing forest productivity by manipulating thinning regimes [11].

### 5.1. Modeling Approach for Predicting Overcompensation

Predicting overcompensation could be based on analytical results using empirical data. However, the time required to obtain field experimental results could be very long, usually well beyond the average professional career. Therefore, research on predicting overcompensation can only be based on available historical datasets (e.g., Figure 1 and Figure 2).

As pointed out in [37], “what makes the science of forestry necessary are the limitations of the traditional empirical approach and recent modeling efforts”. Modeling is a complementary tool of speeding up research for predicting overcompensation. We demonstrated two different approaches that are based on two different mechanisms under the CG conceptual framework. In the TAG model, a top-down approach based on LHT was employed to simulate how individual trees allocate available energy to growth, reproduction, maintenance, and reserves to maximize their inclusive fitness for reproductive success. In the TreeCG model, a bottom-up approach based on actual growth relationships observed from natural stands was used to simulate the consequences of forest redistributing freed resources like space and nutrients from dead trees to survived trees to maximize the utilization of available resources on site. Both models indicated that similar patterns of post-thinning stand dynamics could be expected, including the transition from under compensation to exact compensation and to the overcompensation of a full CG process. However, the TreeCG model explains how overcompensation is generated, whereas the TAG model explains why.

The TAG and TreeCG models were both developed using the concept of CG that was introduced to explain the observed overcompensation phenomenon from the Shawnigan Lake trial that was unable to be explained by conventional thinning theory. The prediction from the two models successfully confirmed the CG concept and the possibility of overcompensation occurring in post-thinning stand dynamics and thus further verified the reconciliation of overcompensation with conventional thinning theory. In other words, the CG concept can expand existing thinning theory to explain both short- and long-term observations and thus resolve the silviculture puzzle.

To make the model practical in operation, in addition to model validation with field observations like TreeCG, methods of calibration to given species/forest types, geographical regions, and site conditions must be developed, as have also been described in the TreeCG paper [32].

### 5.2. Pros and Cons of Overcompensation

Thinning is the practice of selectively removing trees from a forest stand to reduce competition for resources such as light, water, and nutrients. Some key benefits have been identified through enhanced growth rates due to increased access to the essential resources for surviving trees [38], improved tree quality through allowing surviving trees to develop larger crowns and stronger trunks [39], increased stand economic value by promoting the growth of high-value species and improving overall health [40], enhanced carbon storage in biomass [10] and soil [41], a reduced risk of pests and diseases spread in overcrowded conditions, and enhanced wildlife habitats through the creation of a more diverse and open habitat for supporting a wider range of species.

In addition, overcompensation has been widely observed in many species of plants and animals, and different industries have benefitted from this phenomenon tremendously in two aspects: enhanced productivity and reduced costs for production, typically in agriculture (e.g., cotton, beans, and fruit vegetables) and farm animal production or husbandry (e.g., livestock and fisheries) (see a summary from [12]). In forestry, however, overcompensation was not considered as the norm and was often ignored as an “out-ot-the-ordinary” phenomenon due to it being incongruent with the conventional understanding of the thinning effect. The introduction of the CG concept reconciled overcompensation with conventional thinning theory and allowed the development of a model for predicting the occurrence of overcompensation.

Despite the fact that all the mechanisms of overcompensation have still not been fully uncovered, the confirmation of the phenomenon of overcompensation in post-thinning stand dynamics appears to be good news for reaching the goal of enhanced productivity and biomass carbon pooling through sustainable forest management; however, it may alter the temporal pattern of fuel accumulation.

## 6. Conclusions and Recommendations

The take-home messages from this systematic investigation are as follows: (1) five long-term PCT datasets confirmed that overcompensation is common in Canadian forests; (2) the conceptual framework of CG can reconcile different observations from both short- and long-term PCT experiments; (3) the TAG model theoretically proved that overcompensation is an expected phenomenon in long-term post-thinning stand dynamics; and (4) the TreeCG model confirmed that overcompensation can be predicted from growth relationships from natural stands.

As a result, further refinement of the models for regional major tree species or forest types could enable forest managers and researchers to explore the optimal thinning strategies and optimal plantation densities for areas under management, and support decisions in forestry practice, with a view to reaching the goals of maximizing forest productivity and utilization while maintaining resource sustainability for the next generations.

## Data Availability

Permission to use the empirical datasets in this study was obtained from the data providers; further inquiries can be directed to the corresponding authors.
